# Digital Health Needs and Preferences During Pregnancy and the Postpartum Period: Mixed Methods Study

**DOI:** 10.2196/48960

**Published:** 2024-01-12

**Authors:** Natalie Henrich, Alison Brinson, Alyssa Arnold, Hannah R Jahnke

**Affiliations:** 1 Maven Clinic New York, NY United States; 2 Department of Anthropology, University of North Carolina at Chapel Hill Chapel Hill, NC United States

**Keywords:** digital health, perinatal, pregnancy, postpartum, interviews, survey, user needs, patient centered, mixed methods

## Abstract

**Background:**

Digital health is increasingly used to meet the needs of perinatal people, with estimates of pregnancy-related internet use ranging from 90% to 97% of pregnant people. As digital health takes on greater importance during the perinatal period, it is essential that providers and developers of digital health content understand why perinatal people use these resources and the features that enhance their experience. However, gaps remain in understanding the content that is most helpful and how the platforms are navigated. Learning directly from perinatal people about their needs will help ensure alignment between perinatal needs and available content.

**Objective:**

This formative study aims to identify the reasons why perinatal people use digital health resources; the features of the digital health platforms that are of greatest importance to them; and how these differ by perinatal stage (pregnancy vs post partum), mental health conditions, parity, and demographics (race and ethnicity).

**Methods:**

This mixed methods study used interviews; surveys; and secondary data on demographic, health, and pregnancy characteristics to identify the digital health needs and preferences of pregnant and postpartum people who used the Maven digital health platform in the United States during their pregnancy or postpartum period. The interviews informed the content of the surveys and provided additional insights and examples for interpreting the survey results. The surveys were used to collect data from a sample of Maven users, and the results were linked to the secondary data set. The interviews were thematically analyzed, and survey data were analyzed using descriptive statistics and stratified by parity, race, and mental health status.

**Results:**

Overall, 13 people were interviewed (including n=4, 31% pilot interviews), and 147 pregnancy and 110 postpartum survey respondents completed the surveys and had linkable secondary data. Top reasons for using digital health resources during pregnancy were to (1) know what is normal or typical during pregnancy, (2) have access to a health care provider when needed, and (3) know how the baby is developing. Top reasons for postpartum use were to (1) help with breastfeeding, (2) know what normal baby development is, and (3) help with the baby’s health issues. Top platform features during pregnancy and the postpartum period were (1) credible and trustworthy information and providers, (2) nonjudgmental information and support, and (3) no cost to the user. In general, more reasons for using digital resources were identified as extremely important during pregnancy compared with post partum. The results showed minor variations across strata.

**Conclusions:**

This formative research found minor differences in digital resource needs and preferences across user characteristics among perinatal people in the United States. Future work should examine whether there are variations in interests within topics by user characteristics, which may provide additional opportunities to better meet user needs.

## Introduction

### Background

*Digital health*, which refers to digital health applications, ecosystems, and platforms [1-4], is increasingly used to meet the needs of perinatal people [5-8], with estimates of pregnancy-related internet use ranging from 90% to 97% of pregnant people [9-13]. Digital health is expected to have growing importance because of its potential to provide health prevention, consultation, treatment, and management while simultaneously providing an opportunity to reduce costs and improve access to care and patient satisfaction, which together may lead to better informed and more engaged patients [1,4,14,15].

To date, research on perinatal digital health needs falls into 2 categories: self-report via surveys or qualitative data collection (interviews and focus groups) and content analysis of perinatal digital forums. Previous research has shown that access to perinatal digital health information provides reassurance and support [5,16,17]. Both types of studies have found that most people use digital resources during pregnancy to access information on maternal health (eg, pregnancy ailments, pain and complications, and health and nutrition during pregnancy) and during the postpartum period to access information on baby-related topics such as infant sleep and feeding (especially breastfeeding) [18,19]. Self-report studies have also indicated interest in childbirth, fetal and infant development, and infant illnesses, and studies of web-based forums have revealed interest in people and relationships [5,10,11,18-21]. The relative interest in these topics and the specific content within them vary throughout the perinatal period [17,19,20]. Research has also found that a positive experience using digital health resources is characterized by being easy to access and navigate, providing trustworthy and unbiased information, and being customized (eg, trackers and localized information) [4,22,23]. Perinatal digital health users specifically value an experience that provides information that is proactively delivered; offers immediate access; and provides information that is practical and concise, entertaining, and reassuring [5,24,25].

As digital health takes on greater importance during the perinatal period, it is essential that providers and developers of digital health content understand why perinatal people use these resources and what contributes to a positive user experience [26]. The information sought by perinatal people differs from the information that health care providers prioritize and offer. For example, postpartum people seek information on sleep, emotional changes, and breastfeeding, whereas providers focus on medical issues such as bleeding and infection [27,28]. Although there is a growing body of literature, there remain gaps in understanding of the content that is most helpful and how the resources are navigated [5]. There is a demand for more detailed analyses, including which digital resources are most helpful [16], with a particular need to understand what is valued during the postpartum period [18]. Research is needed on digital health needs and experience among perinatal people with mental health conditions, especially pre- and postpartum anxiety [20], and more insights are needed on barriers to and facilitators of using digital health, especially with newer technologies such as apps [4,20]. Learning directly from perinatal people about their needs will help ensure alignment between perinatal needs and available content [18,29]. The design of digital health content should be directly informed by individuals and their experiences. However, the research on patients’ user experience of digital health is limited, especially during the perinatal period [4].

### Objectives

This mixed methods study, which used formative interviews and surveys, investigated the reasons why pregnant and postpartum people use digital health resources and what contributes to a positive user experience. Using a national sample of pregnant and postpartum users of Maven [30], a comprehensive reproductive and family digital health platform that includes patient education, care coordination, and provider services, this formative study identified how needs and preferences differ by perinatal stage (ie, pregnancy vs post partum), mental health conditions, parity, and demographics (race and ethnicity). The results from this study can be used to inform a user-centered approach to developing the content and design of digital health resources and improve the alignment between resources and users. In recognition of the fact that patients have diverse gender identities, we have used gender-inclusive language throughout this paper. If a participant described themselves using gendered language (ie, “as a working mother...”), we used their terminology. When describing the results of previous research, we used the terminology used by the study investigators.

## Methods

### Study Design

**Figure 1:**
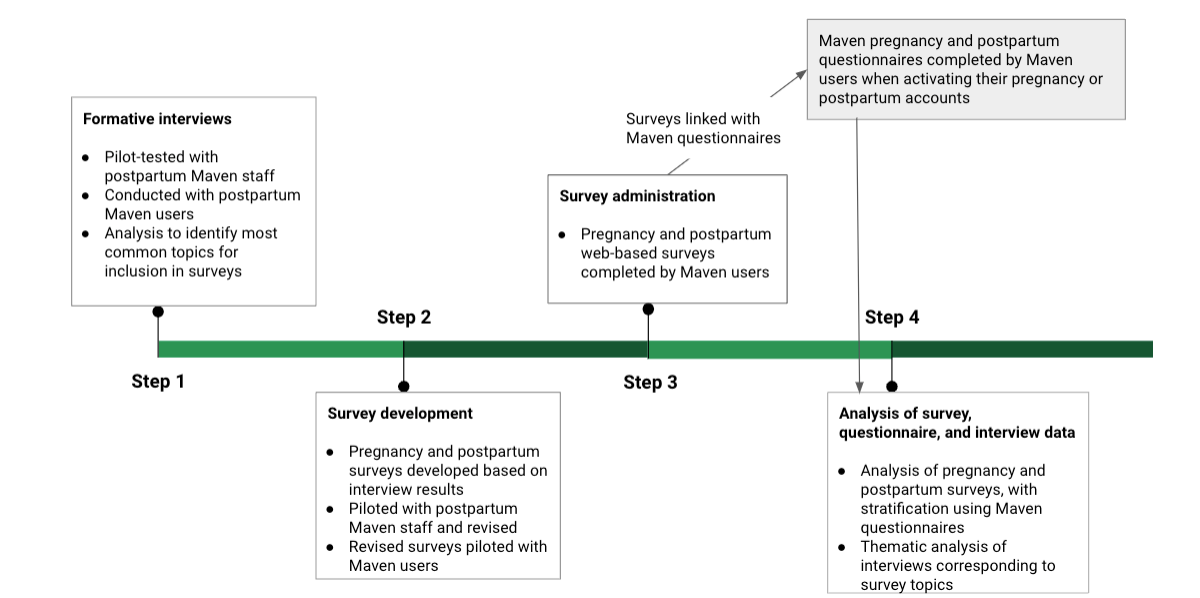
Study process.

In this mixed methods study, we identified the digital health needs and preferences of pregnant and postpartum people who used the Maven digital health platform during their pregnancy or postpartum period. The study used formative interviews to inform the content of surveys and provide additional insights and examples for interpreting the survey results. Surveys were used to collect data from a sample of Maven users that could be linked to a secondary data set of demographic, pregnancy, and health characteristics in the Maven platform (Figure 1).

### Digital Platform Background

Maven was developed in 2014 as a digital platform to support perinatal people and their families. Users have a primary point of contact called a care advocate, an allied health professional (eg, social worker or nurse) who works with the user to navigate the services and resources on the platform. Services and resources include web-based classes; educational materials; and internet-based appointments with a diverse team of health care providers, including obstetrician-gynecologists, mental health professionals, midwives, and doulas. Access to Maven is a sponsored benefit through an employer or the health plan of the user or their partner. The study investigators are Maven employees of the Maven clinical research team. When activating their Maven accounts, users consent to the use of their contact information by Maven for various reasons, including research. The investigators only have direct contact with Maven users or access to identifiable data within the scope of specific research projects, in accordance with institutional review board (IRB) approvals, and in concordance with Health Insurance Portability and Accountability Act regulations. Access to user data is minimized by requesting recruitment lists and platform-based data from the Maven product analytics team, which deidentifies the information before sharing it with the research team. To recruit Maven users for interviews and the survey, the recruitment parameters were sent to the product analytics team, which generated a list of deidentified IDs of users who met the parameters. This list of deidentified IDs was sent to the Maven team responsible for direct communication with users, which sent out recruitment emails (using email addresses linked to the deidentified IDs).

### Formative Interviews

In September 2022 and October 2022, semistructured interviews with postpartum people were conducted virtually via Zoom (Zoom Video Communications) to identify the reasons why perinatal people use any digital health resources, not just Maven. To help encourage participants to think about digital use broadly, at the start of the interview, they were asked to list all the digital resources used during their pregnancy and the postpartum period, and throughout the interview, they were reminded to think about digital resources in general. The first part of the interview was open-ended, and participants were prompted not only to describe the reasons why they used digital resources but also to identify additional reasons for which they would like to use them. In the second part of the interview, participants were shown (via screen sharing on Zoom videoconferencing) a list of reasons why digital resources could be helpful during the perinatal period. Participants were asked whether they had used a digital resource for each individual reason or whether they would like to use a digital resource for each individual reason in the future. Finally, participants were asked about the importance of digital health resources being inclusive of their entire identity, which was explained as including anything that the participant considered part of their identity, such as race, gender, sexual identity, geography, social or economic status, and mental and physical health conditions, and to describe their experience with (lack of) inclusion.

Pilot interviews were conducted with 4 postpartum Maven staff members who had used Maven during pregnancy and the postpartum period and were post partum at the time of the interview. The interview guide was modified based on their feedback and the interviewer’s learnings from conducting the pilot interviews. Following piloting, interviews were conducted with 9 Maven users, all of whom were post partum so that they could speak to their digital needs and preferences throughout the perinatal period. Participants were recruited with an emphasis on including people from a range of ethnicities and races, health conditions, and parity. Participants were recruited via email. Interviews lasted approximately 1 hour, were conducted via Zoom, and were recorded. An interviewer and a notetaker were present, and transcripts were autogenerated on Zoom. Participants (Maven staff and nonstaff) consented to the interview, had the option of having the video on or off, and could decline to have the interview recorded (one participant declined, and we relied on detailed notes for that interview). The consent form was included in the recruitment email and reviewed at the start of the interview, and consent was provided verbally. Participants were given a US $50 Amazon gift card as a *thank you* for their participation.

The prevalence of each reason for using digital resources was calculated by counting the number of interviews (Maven staff and nonstaff) in which the reason was mentioned as important. The reasons that were mentioned as important in the most interviews were included in the survey.

### Survey Development

In addition to the semistructured interviews, separate quantitative surveys were developed to inquire about digital resource use during pregnancy and the postpartum period. Through the qualitative analysis, it emerged that participants were describing 2 aspects of digital resource use: the information or support they were seeking and features of the digital health platforms. Consequently, the survey contained 2 sections, each addressing one of these aspects. To focus on common needs and remove outliers that were mentioned by only 1 or 2 participants, reasons for using digital resources and features affecting experience were excluded from the surveys if they were cited by less than one-third of the interview participants. In each section of the survey, participants indicated the importance of each item on a Likert scale that included a description of each option (*a little important or doesn’t matter to me*—*I don’t care much about this*, *important*—*I would like to have this but I’m OK without it*, *very important—I care about this a lot but it’s not absolutely necessary*, and *extremely important—a “must have”*) and subsequently picked the 5 most important items (not ranked). We opted to allow participants to select up to 5 items as a means to balance the tension between the high number of reasons why pregnant and postpartum people are turning to digital resources and the need to prioritize to inform actionable decisions about what content to provide in these resources. To reduce the potential bias of basing responses on their use of Maven, the instructions in the survey reminded respondents to think holistically about digital health resources. The following is an example of the instructions from a section of the pregnancy version of the survey: “Thinking about the reasons you might use any digital resource during pregnancy, rate how much each of these reasons matter to you. Digital resources include websites, apps, videos, blogs, search engines, etc.”

The surveys were piloted with 4 Maven staff members who had used Maven during pregnancy and the postpartum period and were post partum at the time of piloting. The wording and format were modified based on their feedback. The revised surveys were piloted with 4 Maven nonstaff users. During piloting, we identified that nearly all items were selected as *very important*. To minimize ceiling effects, the response options were revised to provide more response options that reflected higher levels of importance and add definitions (eg, *extremely important: a must have*). We also opted to reverse the order of the options (with *a little important or not important to me* as the first option rather than the last). In addition, we included a section for selecting the 5 most important reasons for use and platform features, which would provide information on the relative importance of the items if we continued to have a ceiling effect.

On the basis of feedback from the staff and nonstaff member pilots, we added 2 items that had not emerged in the interviews: using digital resources to help with formula feeding and feeding solid foods. These were added as participants felt that the survey was not inclusive or relevant to the entire postpartum period if it only asked about breastfeeding.

The survey was built in SurveyMonkey (SurveyMonkey Inc) using a Health Insurance Portability and Accountability Act–compliant account.

### Survey Administration

Survey respondents were recruited from current Maven users. To be eligible for the pregnancy survey, users had to be between 0 and 6 months post partum and have had a Maven account during pregnancy. To be eligible for the postpartum survey, users had to be 3 to 12 months post partum and have a Maven account during the postpartum period. Recruitment was via email, which included a link to the survey. Each survey link was associated with the user’s unique Maven identifier to link the survey responses to user demographic characteristics previously recorded on the Maven platform (described in the *Secondary Data* section). A reminder email was sent 1 week after the initial email to anyone who had not clicked through to the survey. The survey remained open for 2 weeks from the time of the initial email. A consent form appeared before the start of the survey, and consent had to be provided digitally to access the survey. Participants were entered into a draw for a chance to win 1 of 5 US $100 Amazon gift cards per survey (5 for the pregnancy survey and 5 for the postpartum survey). Data were collected from January 18 to 29, 2023.

### Secondary Data

Maven users are encouraged but not required to fill out a questionnaire when they activate their account. The Maven pregnancy questionnaire collects data on physical and mental health, pregnancy conditions, demographics, and parity. The Maven postpartum questionnaire collects information on demographics, mode of delivery, and previous pregnancy conditions. For survey participants who completed the Maven questionnaires, we linked their survey responses with their Maven questionnaire data using their unique Maven identifier. Maven users consent to the use of their Maven questionnaires for research when they activate their accounts, and the research team does not have access to any identifying information.

### Analyses

#### Survey Analysis

**Figure 2:**
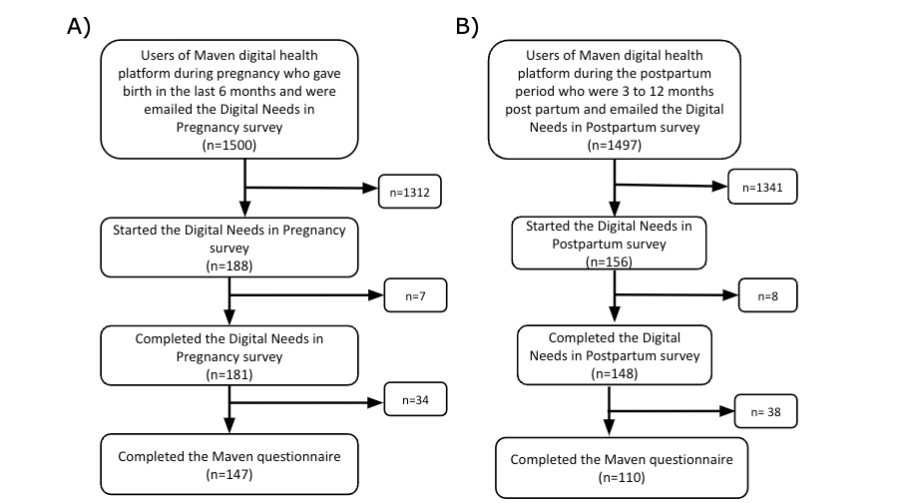
Flowchart of the pregnancy survey respondents (A) and postpartum survey respondents (B) included in the survey analysis.

Respondents who completed the survey and a Maven questionnaire were included in the survey analysis. Figure 2 shows the pathway for inclusion in the analysis, from eligibility to be recruited to starting and completing the survey and completing a Maven questionnaire. The quantitative data from both the pregnancy and postpartum surveys were assessed primarily through the evaluation of descriptive statistics. Chi-square and Fisher exact tests were used to assess differences among pregnancy items, postpartum items, and user experience features by parity, presence of any mental health conditions, and race and ethnicity. A user was considered to have a mental health condition if they reported any of the following: a history of anxiety, depression, perinatal mood disorder, or high pregnancy-related anxiety. A history of anxiety or depression was assessed in the Maven questionnaires using the following question—“Do any of these conditions apply to you or did they in the past?”—with the selection of *Anxiety or depression* from a list of conditions. Experience of perinatal mood disorder was assessed using the following question—“Have you experienced any of the following during this pregnancy?”—with a selection of *Perinatal mood disorder*. Pregnancy-related anxiety was assessed on a 5-item Likert scale in response to the following question—“On a scale of 1-5, how anxious are you feeling about your pregnancy?”—with responses of 4 (*very*) or 5 (*extremely*) indicating the presence of pregnancy-related anxiety. For analyses stratified by race and ethnicity, we recategorized individuals who identified as Hispanic, Black, Asian or Pacific Islander, or multiracial into 1 category labeled *non-White* because of our small sample of people of racial and ethnic minorities. Consistent with the study’s aim of identifying differences in needs and preferences between respondents with these characteristics, *P* values were calculated to determine whether the differences were statistically significant (with results considered to be statistically significant if the *P* value was <.05). Statistical analyses were conducted using RStudio (Posit Software, PBC).

#### Interview Analysis

The interviews were analyzed thematically using a framework analysis [31-36]. Themes were identified deductively based on the interview guide and inductively based on the messages that emerged from the interviews. Themes and their definitions were reviewed by all team members who participated in the interviews (as interviewers or notetakers) and were modified based on group discussion and consensus. The analysis table was populated based on the notes taken during the interviews and subsequent analysis discussions. Audio recordings and transcripts were referenced if the notes were unclear or lacked sufficient details to populate the analysis table. All entries in the analysis table were reviewed by NH, who attended all but one of the interviews. Any information that was missing or potentially miscategorized in the table was flagged for review by the person who entered the information and, if necessary, discussed to reach a consensus. To better understand the experiences and perspectives associated with the topic of each of the items in the quantitative surveys, the corresponding interview themes were summarized, and representative anecdotes or quotes were selected. All quotes were verified using the audio recordings. Quotes and anecdotes are cited using the interview participant number (eg, P1 refers to participant 1), and it is specified whether the participant was from the pilot interviews (eg, Pilot P1 refers to the first interview among the pilot participants).

### Ethics Approval

The study protocol was approved by the WCG IRB (study 1338443). The use of secondary data was deemed exempt by the WCG IRB under 45 Code of Federal Regulations § 46.104(d)(4).

## Results

### Survey Sample Characteristics

In total, we had 147 pregnancy survey respondents and 110 postpartum survey respondents with complete Maven questionnaires (Table 1 and Figure 2). All survey responses were collected within 48 hours of sending the initial recruitment email. The average age of the respondents was 32.8 (SD 4.6) years and 32.7 (SD 3.5) years for pregnancy and postpartum survey respondents, respectively. On the basis of those who reported their race and ethnicity, our sample was comparable with the overall US population in terms of the percentage of people identifying as non-Hispanic White (134/257, 52.1%) and multiracial (5/257, 1.9%), with an overrepresentation of people identifying as Asian (31/257, 12.1%) and an underrepresentation of people identifying as Black (4/257, 1.6%) or Hispanic (16/257, 6.2%) [37]. A total of 44.9% (66/147; pregnancy survey) and 51.8% (57/110; postpartum survey) of the respondents had household incomes of >US $100,000. Approximately 28% (72/257) of the sample reported a history of a mental health condition (Table 1).

**Table 1:** Characteristics of respondents who took the pregnancy survey and the postpartum survey.

Demographic characteristics^a^			Pregnancy survey respondents (n=147)		Postpartum survey respondents (n=110)
Age (y), mean (SD)			32.8 (4.6)		32.7 (3.5)
**Race and ethnicity, n (%)**					
	Hispanic	8 (5.4)		8 (7.3)	
	Non-Hispanic Asian	21 (14.3)		10 (9.1)	
	Non-Hispanic Black	3 (2)		1 (0.9)	
	Non-Hispanic multiracial or American Indian	3 (2)		2 (1.8)	
	Non-Hispanic White	77 (52.4)		57 (51.8)	
	I prefer not to say	35 (23.8)		17 (15.5)	
**BMI (kg/m^2^), n (%)**					
	≤18.5	4 (2.7)		0 (0)	
	18.5 to 24.9	76 (51.7)		49 (44.5)	
	25 to 29.9	30 (20.4)		15 (13.6)	
	≥30	29 (19.7)		15 (13.6)	
Social Vulnerability Index^b^ (high), n (%)			10 (6.8)		2 (1.8)
**Household income (US $), n (%)**					
	20,000-34,999	1 (0.7)		1 (0.9)	
	35,000-49,999	3 (2)		2 (1.8)	
	50,000-74,999	7 (4.8)		3 (2.7)	
	75,000-99,999	18 (12.2)		9 (8.2)	
	≥100,000	66 (44.9)		57 (51.8)	
	I prefer not to say	27 (18.4)		10 (9.1)	
Nulliparous (yes), n (%)^c^			97 (66)		58 (80.6)
**Mental health history, n (%)^c^**					
	Anxiety	33 (22.4)		18 (25)	
	Depression	14 (9.5)		4 (5.6)	
	Perinatal mood disorder	1 (0.7)		2 (2.8)	

^a^Variables with <15% of missing data: age, race, social vulnerability, BMI (pregnancy survey respondents), mental health history (pregnancy survey respondents), and parity (pregnancy survey respondents). Variables with 15% to 30% of missing data: household income and BMI (postpartum survey respondents). Variables with 30% to 40% of missing data: parity (postpartum survey respondents) and mental health history (postpartum survey respondents).

^b^Measured using the Centers for Disease Control and Prevention Social Vulnerability Index.

^c^A total of 65.5% (72/110) of postpartum survey respondents provided parity or mental health data. Therefore, the sample size for those variables is 72.

### Reasons for Use of Digital Resources During Pregnancy

On the basis of the survey responses, the most important reasons for using digital resources during pregnancy were to know what is normal or typical during pregnancy, have access to a health care provider when needed, and know how the baby is changing and developing during pregnancy (Table 2).

**Table 2:** Reasons identified as extremely important when using digital health resources during pregnancy by parity, mental health status, and race and ethnicity (N=147).

Reasons for using digital health resources during pregnancy	Overall, n (%)	Parity			Mental health				Race and ethnicity^a^		
		Parous (n=50), n (%)	Nulliparous (n=97), n (%)	*P* value	No mental health history (n=105), n (%)	Mental health history (n=37), n (%)	*P* value	People of racial and ethnic minorities (n=35), n (%)		Non-Hispanic White (n=77), n (%)	*P* value
To know what is normal or typical during pregnancy	95 (64.6)	24 (48)	71 (73.2)	.004	68 (64.8)	24 (64.9)	>.99	18 (51.4)		54 (70.1)	.17
To have access to a health care provider when I need them	84 (57.1)	26 (52)	58 (59.8)	.49	57 (54.3)	24 (64.9)	.38	22 (62.9)		37 (48.1)	.10
To know how my baby is changing and developing during pregnancy	76 (51.7)	20 (40)	56 (57.7)	.06	58 (55.2)	16 (43.2)	.25	18 (51.4)		41 (53.2)	>.99
To help me manage discomfort during pregnancy	61 (41.5)	19 (38)	42 (43.3)	.69	44 (41.9)	16 (43.2)	>.99	18 (51.4)		30 (39)	.18
To become more empowered to speak up for myself during pregnancy and labor	56 (38.1)	16 (32)	40 (41.2)	.38	41 (39)	13 (35.1)	.78	14 (40)		28 (36.4)	.68
To explore my options for labor and delivery	55 (37.4)	14 (28)	41 (42.3)	.14	42 (40)	11 (29.7)	.33	14 (40)		28 (36.4)	.68
To help me manage anxiety or depression	55 (37.4)	19 (38)	36 (37.1)	>.99	37 (35.2)	15 (40.5)	.74	21 (60)		24 (31.2)	.003
To help me manage my health problems (chronic, ongoing, or related to pregnancy)	45 (30.6)	17 (34)	28 (28.9)	.62	32 (30.5)	12 (32.4)	>.99	18 (51.4)		17 (22.1)	.002
To get additional information or clarification on things my doctor tells me	44 (29.9)	15 (30)	29 (29.9)	>.99	32 (30.5)	10 (27)	.82	13 (37.1)		18 (23.4)	.13
To know the size of my baby throughout pregnancy	35 (23.8)	14 (28)	21 (21.6)	.49	29 (27.6)	6 (16.2)	.23	13 (37.1)		16 (20.8)	.07
To help me with healthy eating during pregnancy	32 (21.8)	7 (14)	25 (25.8)	.16	27 (25.7)	3 (8.1)	.04	11 (31.4)		13 (16.9)	.09
To help me with being physically active during pregnancy	30 (20.4)	9 (18)	21 (21.6)	.78	26 (24.8)	4 (10.8)	.11	9 (25.7)		14 (18.2)	.40
To get recommendations of things to buy to care for my baby or myself	25 (17)	7 (14)	18 (18.6)	.66	20 (19)	4 (10.8)	.36	9 (25.7)		12 (15.6)	.24
To connect with a community of people who are in the same stage of pregnancy as me	17 (11.6)	6 (12)	11 (11.3)	>.99	11 (10.5)	6 (16.2)	.54	4 (11.4)		10 (13)	>.99

^a^Respondents who selected *I prefer not to say* for race and ethnicity were not included in this comparison.

In interviews, participants described the role that digital resources play in meeting these needs (Table 3), and they emphasized the importance of these resources during their first pregnancy (Pilot P1 and P2). Among the information they sought about what is normal, participants wanted to know what they would encounter during each phase of labor and delivery and were looking for information such as how long to expect to push. A participant described learning about the phases during a digital birth-planning appointment:

[The digital appointment with the birth planning specialist] was very helpful to know what to expect at the hospital during birth, and actually I feel like they were more helpful than the in person doula that I hired...I remember them stepping through the birth process. You know, active labor and transition, the pushing and talking about the ring of fire.

P5

**Table 3:** Illustrative quotes for using digital resources (pregnancy and postpartum interviews).

Reasons for using digital health resources			Illustrative quote
**During pregnancy**			
	To know the size of my baby throughout pregnancy	“What is baby now today? Let’s see. I will also open the app up and see. So that was a good thing as well. So this is like ‘What vegetable is your baby right now?’” (P2)	
	To help me manage discomfort during pregnancy or postpartum	“[The digital resource helped with] cracked and bleeding nipples” (P9).	
	To become more empowered to speak up for myself during pregnancy and labor	“I read a ton of articles [online] because I felt like otherwise it was like the blind leading the blind. My husband had never done that before. I’m an only child like my mom was going to be there, but she hadn’t done it in thirty years. So it was kind of like, Okay, Who in this room knows what they’re doing? The answer was going to be nobody...So I read a lot just to know these are all the possible outcomes. This is how it might go. These are the things that I’m going to be asked, and I wanted to know, while I’m in a calm and sound state of mind, like is my answer Yes? Is my answer No? What are the implications if I say yes or no. And then [I can] communicate that to my partner and my mother. So if they needed to advocate for me [they could]” (P4).	
	To explore my options for labor and delivery	“[Exploring my labor and delivery options] I would say [getting support on Maven] helped a little but that was more so because of me, and I didn’t look into it as much as I should have...Because, I thought I would have a natural birth. But I ended up having a C section, but just not knowing what to expect, and more so it was on the process of like what an induction is, and like how your body would look like afterwards, because I didn't get to in my entire pregnancy. But all the drugs they pumped into you to induce you caused me to blow up after, and I just didn't expect that, no one tells you, right? They just tell you to consent, and you say yes, so like all that, I wish I knew. But I don't think that I looked into it as much, because I just didn't know” (P8).	
**During the postpartum period**			
	To help me with feeding my baby breast milk	“I didn’t see lactation consulting in person until after birth, which was at the pediatricians office. So it’s nice to have that, to be able to talk to one [online] beforehand, to know what to prepare for. And then also I talked to one after as well, after giving birth. That was super helpful” (P5).	
	To help me with my baby’s health issues	“[My baby] was very colicky, I took an [online] appointment and showed the doctor what was happening, so they also gave me a hint she might have a milk allergy, [and said to] check with your doctor and check the stools and all” (P2).	
**During pregnancy or the postpartum period**			
	To know what is normal or typical during pregnancy or postpartum	“[Getting digital help on my baby’s sleep] helped a lot, because I had a lot of questions about what’s normal, what’s not normal and they answered everything. It made me feel like I wasn't doing something bad” (P8).	
	To know how my baby is changing and developing during or after pregnancy	“I had like three apps on my phone to track [how my baby was changing] like every week” (P4).	
	To have access to a health care provider when I need them	“It wasn’t just 8 to 5 that I could access someone...The sleep consultant was like you can just message me any time, and I’ll respond and help you real time with what you need. That’s something that you can’t get with any in person care provider” (P3).	
	To help me with being physically active during pregnancy or the postpartum period	“I was trying to be physically active during pregnancy but then there were times where I stopped because of the spotting. They didn’t know what was going on. Then I found out it was okay. But then the last trimester it got tougher since I was tired a lot and having pain...It would have been nice to see what I could do, like modifications and exercises I could do around the pain I was having” (P5).	
	To help me with healthy eating during pregnancy or the postpartum period	“Just offering ideas of foods, more nutritious foods to eat, healthier options, because you can look around on the Internet but just talking to a person who is, you know, that’s their specialty, and you can give them personal information about yourself so they can help you” (P9).	
	To help me manage anxiety or depression	“I was in so much pain and I thought maybe [the baby will] be also in pain, or somehow it will affect the baby, because I’m not happy. So then I talked to [an online] doctor, and one of the doctors was very good and she talked to me more than the time of appointment, and she was very kind, and she said, I know how you are feeling, but she gave me so much support, emotional support, and she said, ‘You can come to talk to me anytime.’...So she then she assured me, ‘Everything is fine, and you don’t need to worry’” (P2).	
	To help me manage my health problems (chronic, ongoing, or related to pregnancy or postpartum)	“I have arthritis and Hashimoto. So two autoimmune diseases. So when I got pregnant, I had a lot of concerns about that, and how that would affect my pregnancy, or vice versa” (P8).	
	To get recommendations of things to buy to care for my baby or myself	“I’m a working mother. At the time, you know, working while pregnant, and so I didn’t have time to spend hours and hours going down the rabbit hole of like, this stroller versus that stroller. Like just like, tell me what you recommend, and we’re moving on. So it felt like the research had been done for you” (P4).	
	To connect with a community of people who are in the same stage of pregnancy as me or who have a baby the same age as mine	“I used Reddit honestly before I was telling people I was pregnant...I really kind of used Reddit to ask people different questions early on in my pregnancy like ‘Oh, this happened to me. Is this normal?’ Like I said, it was in the very early stages of before we were talking to anybody, so I didn’t really have anyone to talk to” (P7).	
	To get additional information or clarification on things my physician or pediatrician tells me	“The pediatrician’s really good for, like, is the baby’s body healthy, but they’re not necessarily specifically trained in infant nutrition and infant sleep. They kind of know enough to be dangerous. You know, I would ask some questions like, ‘How do I get my baby sleeping through the night longer?’ And they would say something really old school like ‘Put rice cereal in their bottle,’ and I’m like well, one I’m not even giving a bottle, I’m breastfeeding and two, that’s like not really even something people say anymore. So it’s just like our pediatrician wasn’t super helpful. I totally trust him for everything else. But just not those services” (P3).	

In interviews, participants talked about being able “to count on” accessing health care providers on the internet for urgent situations, such as a participant (P1) who used a digital health care provider when she did not feel her baby kick for a while and wanted to know whether she should go to the emergency room.

Almost all the participants talked about the importance of knowing how their baby was growing and developing during pregnancy. They described wanting to know about the development week by week and found that digital resources could easily provide this information.

In the survey, nulliparous respondents were more likely than parous respondents to find knowing what was normal (*P*=.004) and how their baby was developing (*P*=.06) to be extremely important (Table 2). Compared with non-Hispanic White respondents, respondents identifying as Hispanic or people of racial and ethnic minorities were more likely to report that digital health resources were extremely important for the management of anxiety or depression (*P*=.003) and chronic health problems (*P*=.002). Compared with respondents without a mental health history, respondents with a mental health history were more likely to find it important to receive web-based help with healthy eating during pregnancy (*P*=.04; Table 2).

### Reasons for Use of Digital Resources During the Postpartum Period

On the basis of survey responses, during the postpartum period, the most important reasons for using digital resources were to help with feeding the baby breast milk, know what is normal baby development (eg, developmental milestones), help with the baby’s health issues, and help with feeding the baby solid foods (Table 4).

The reasons for using digital resources during the postpartum period were described in interviews (Table 3). When asked specifically about the use of digital health resources for help with feeding, interview participants primarily spoke about support for breastfeeding. In particular, participants found it valuable to connect with a lactation consultant during pregnancy to prepare for breastfeeding and during the postpartum period for tips that facilitated feeding:

I didn’t see lactation consulting in person until after birth, which was at the pediatrician’s office. So it’s nice to have that, to be able to talk to one [online] beforehand, to know what to prepare for. And then also I talked to one after as well, after giving birth. That was super helpful. Just things I could do for milk supply. And then the one major helpful thing that I learned is about the pump parts where I can just store them in the fridge, and I don’t have to wash it after every time, because it was a hassle, too.

P5

In interviews, participants described using digital resources to know what to expect about life with a baby and to find out whether the things they encountered were normal, such as struggling with the baby’s sleep schedule, and what it is really like to breastfeed. Participants voiced frustration that this type of information was not provided by in-person providers, so they looked for answers on the internet:

They don’t tell you anything [at the hospital]. A big one for me was breastfeeding, like just how complex it is. There’s just so many things in the hospital they don’t tell you anything, either. They just ask you like, “Are you going to breastfeed, yes or no,” and you have to decide on the spot, and, like you don’t know anything about this. After that, coming home, I definitely did research on [a digital platform] and the resources that they had for help.

P8

Participants also wanted digital resources to provide information on what is normal for babies as they grow, including what developmental milestones to expect at each age. A participant described using an app to know what to expect as her baby changed:

[There] is an app that when you put in the baby’s due date, this helps you to see your baby’s development, like milestone-wise. So week by week, you know some of the things to look for. And mainly I use it to see like, when are one of the cranky times coming up because they’re going through a milestone leap where they’re learning, you know lots of new things.

P9

In interviews, participants provided anecdotes about the value of digital resources for helping with the baby’s health issues, especially the ability to access support when it is needed. A new parent described meeting with a digital sleep coach in the middle of the night when her baby would not sleep, a service she said she could not obtain from her in-person providers as they only handled emergencies after hours:

It wasn’t just 8 to 5 that I could access someone...The sleep consultant was like you can just message me any time, and I’ll respond and help you real time with what you need. That’s something that you can’t get with any in person care provider. If you call their after hours number it’s because it’s an emergency right? Not because you are just, like, wondering why your baby is fussy, or awake window, or, you know whatever.

P3

In the survey, nulliparous respondents were more likely than parous respondents to find it extremely important that digital resources help with feeding their baby solid food (*P*=.01) and with their own health problems (*P*=.06; Table 4). Respondents identifying as Hispanic or people of racial and ethnic minorities were more likely than non-Hispanic White respondents to report that it is extremely important that digital resources can be used to connect them with a community of people who have a baby of the same age (*P*=.02) and to obtain recommendations of things to buy to care for their baby or themselves (*P*=.08). Respondents with a mental health history were more likely than those without a mental health history to find it important to obtain web-based help with breastfeeding their baby (*P*=.01; Table 4).

**Table 4:** Reasons identified as extremely important when using digital health resources during the postpartum period by parity, mental health status, and race and ethnicity (N=110)^a^.

Reasons for using digital health resources during the postpartum period	Overall, n (%)	Parity				Mental health				Race and ethnicity^b^		
		Parous (n=14), n (%)	Nulliparous (n=58), n (%)	*P* value	No mental health history (n=49), n (%)		Mental health history (n=20), n (%)	*P* value	People of racial and ethnic minorities (n=21), n (%)		Non-Hispanic White (n=57), n (%)	*P* value
To help me with feeding my baby breast milk	64 (58.2)	6 (42.9)	37 (63.8)	.12	23 (46.9)		17 (85)	.01	12 (57.1)		34 (59.6)	>.99
To know what is normal baby development (milestones)	54 (49.1)	7 (50)	30 (51.7)	.89	27 (55.1)		9 (45)	.51	14 (66.7)		25 (43.9)	.12
To help me with my baby’s health issues	54 (49.1)	6 (42.9)	32 (55.2)	.38	24 (49)		12 (60)	.65	13 (61.9)		27 (47.4)	.30
To help me with feeding my baby solid foods	53 (48.2)	4 (28.6)	36 (62.1)	.01	25 (51)		12 (60)	.78	12 (57.1)		25 (43.9)	.43
To help me manage anxiety or depression	45 (40.9)	5 (35.7)	22 (37.9)	.77	15 (30.6)		10 (50)	.24	8 (38.1)		26 (45.6)	.71
To help me with feeding my baby formula	30 (27.3)	2 (14.3)	18 (31)	.20	13 (26.5)		6 (30)	>.99	7 (33.3)		16 (28.1)	.87
To get additional information or clarification on things my physician or pediatrician tells me	29 (26.4)	4 (28.6)	14 (24.1)	>.99	11 (22.4)		6 (30)	.78	5 (23.8)		15 (26.3)	>.99
To help me with my health problems (chronic, ongoing, or related to pregnancy or postpartum)	22 (20)	0 (0)	12 (20.7)	.06	7 (14.3)		4 (20)	.72	4 (19)		11 (19.3)	>.99
To help me with being physically active after my baby is born (postpartum exercise)	18 (16.4)	4 (28.6)	10 (17.2)	.47	8 (16.3)		5 (25)	.50	3 (14.3)		10 (17.5)	>.99
To help me with healthy eating after my baby is born	16 (14.5)	2 (14.3)	10 (17.2)	>.99	9 (18.4)		2 (10)	.48	4 (19)		6 (10.5)	.44
To help me with losing weight after my baby is born	12 (10.9)	1 (7.1)	7 (12.1)	>.99	5 (10.2)		2 (10)	>.99	4 (19)		7 (12.3)	.47
To obtain recommendations of things to buy to care for my baby or myself	11 (10)	1 (7.1)	9 (15.5)	.67	6 (12.2)		4 (20)	.47	4 (19)		3 (5.3)	.08
To connect with a community of people who have a baby the same age as mine	9 (8.2)	0 (0)	6 (10.3)	.33	4 (8.2)		1 (5)	>.99	4 (19)		1 (1.8)	.02

^a^A total of 65.5% (72/110) of postpartum survey respondents provided parity or mental health data.

^b^Respondents who selected “I prefer not to say” for race and ethnicity were not included in this comparison.

The reasons for using digital resources that the fewest survey respondents selected as extremely important during the perinatal period were uses related to general wellness (ie, healthy eating, exercise, and losing weight), connecting with a community in the same perinatal stage, and obtaining recommendations of things to buy to care for their baby or themselves. Notably, these lowest-ranked reasons were still selected by 8.2% (9/110) to 22% (32/147) of the respondents (Tables 2 and 4).

In contrast to the surveys, help with healthy eating was one of the topics that the most interview participants identified as a reason for using digital resources, although it was usually in response to specifically being asked about it in the structured part of the interview. When discussing the use of digital resources for healthy eating, participants tended to refer to access to personalized information such as food recommendations that fit with their dietary restrictions. A participant described why tailored information would be helpful to her on a digital health platform:

Because if I had dietary restrictions I’m going to have to go searching more for that information on my own. [It would have been helpful], I'd say, to come up with a list of foods that would be specific to me, because we didn't do that. I realized that may be a lot to ask of someone. But that's just what I see would be super helpful.

P9

### Importance of Digital Health Platform Features During Pregnancy and the Postpartum Period

The 3 most important features of digital health platforms identified through both the pregnancy and postpartum surveys were credible and trustworthy information and providers, digital resources that are free to the user, and nonjudgmental information and support. Although access to appointments (ie, fast access and access at convenient times) was not among the top 3 most important features, approximately half of the respondents felt that it was extremely important (Table 5). See Multimedia Appendices 1 and 2 for digital health platform features identified as *extremely important* by parity, mental health status, and race and ethnicity during pregnancy and the postpartum period, respectively.

**Table 5:** Digital health user experience features identified as extremely important during pregnancy and the postpartum period.

Digital health platform features	Pregnancy survey respondents (n=147), n (%)	Postpartum survey respondents (n=110), n (%)
Credible and trustworthy information and providers	117 (79.6)	82 (74.5)
Nonjudgmental information and support	97 (66)	58 (52.7)
Digital resources that are free to me	89 (60.5)	60 (54.5)
Fast access to appointments	75 (51)	52 (47.3)
Easy to find information; easy to navigate	74 (50.3)	53 (48.2)
Access to appointments at convenient times	72 (49)	50 (45.5)
Information that is actionable (specific recommendations for what to do)	64 (43.5)	57 (51.8)
Receive fast responses to my digital messages	59 (40.1)	40 (36.4)
Resources that are specific to my needs (personalized)	49 (33.3)	33 (30)
Access to a lot of information on each topic (depth of information)	36 (24.5)	22 (20)
Access to information on a lot of topics (breadth of topics)	35 (23.8)	22 (20)
Consistent care or support from the same people over time on the digital platform	31 (21.1)	33 (30)
Proactive outreach from the digital resource (pushes content to me; provider reaches out to me)	26 (17.7)	12 (10.9)
Care or content that fits with my culture and identity	26 (17.7)	14 (12.7)

Interview participants described why the features of digital health resources were important (Textbox 1). When looking to interact with content that was trustworthy, participants mentioned a range of ways in which they determined whether digital content was credible. A couple of participants explained that they trusted the content if it was consistent with the information they received from their in-person providers. This was done by taking the information from the digital content to the in-person provider for verification or by seeing that the content was consistent with what they had already been told:

Whatever I heard from the [web-based] doctor, it was matching with my experience and my other doctor as well...it builds trust since my doctor is saying the same thing.

P2

So it was like things that the [web-based] practitioner might have suggested or recommended that I would then mention at my doctor’s appointment...kind of to get a check box from my regular doctor, but it was always like, she agreed with what had been communicated.

P4

**Illustrative quotes regarding features of digital health resources (pregnancy and postpartum interviews).**

**Credible and trustworthy information and providers**

- “Sleep schedules, again, with so much information online, you get really caught up in, like, ‘Am I doing the right thing?’ especially in the beginning. I remember one thing. I was struggling with my baby’s sleep schedule. And so I went to this sleep expert from [my digital platform]. And they said that’s normal, like, a baby shouldn’t really have a sleep schedule until six months. They’ll never have one, and it’s like everything online, says the opposite! So just like having information that is accurate and like coming from experts [really helped]” (P8).

**Digital resources that are free to me**

- “But because this service was free to me I felt like ‘well, it’s there, and I might as well use it,’ so I’ve taken advantage of it” (P3).

**Nonjudgmental information and support**

- “And these platforms really help because, one, you’re able to speak to someone different that may not even have your medical history. They don’t have your medical history in front of you. So maybe it’s the medical system, health care system thinking or judging you based on your history, right, rather than just listening to this specific one time incident. So maybe they’re less biased ’cause they just have the one thing they’re looking at” (P8).

**Fast access to appointments**

- “If I’m struggling with a situation that I think is super important with the baby like the baby has a cold and has a runny nose, fever, I’m gonna want whoever is available next” (P9).

**Easy to find information; easy to navigate**

- “[My app] did not help [for finding information on baby’s development] because the information, I guess I didn’t seek it out, but I also didn’t see it readily available” (P9).

**Access to appointments at convenient times**

- “Especially for a new mom, if you’re trying to work around is my baby napping or not, and that’s not always predictable, just to be able to quickly touch base with someone. I used it during pregnancy as well, so I feel it kind of rounds out the care you are receiving from your in person doctor” (P4).

**Information that is actionable (specific recommendations for what to do)**

- “In my state it is legal to end the pregnancy, right? So [the online provider] gave me the support that if you want to go for this, this will happen, or if you go, these are the steps you need to follow, and all...She was telling me all the steps, what I need to do, and what I not to do, and she gave me a support for keeping this pregnancy” (P2).

**Receive fast responses to my digital messages**

- “When I messaged, it wasn’t always [the same person] that responded, it was someone else. But it’s nice to have that, too. Like, someone quickly getting back to me. So I didn’t mind that” (P5).

**Resources that are specific to my needs (personalized)**

- “Because if I had dietary restrictions I’m going to have to go searching more for that information on my own. [And it would be helpful to] come up with a list of foods that would be specific to me, because we didn't do that. I realized that may be asking a lot of someone. But that's just what I see would be super helpful” (P9).

**Access to a lot of information on each topic (depth of information)**

- “I like the options when I went in, and I would read through an article. It’s like, Oh, here’s some other related stuff to this. If you’re still interested. I really like that because there was a couple of times with a few articles where, like I want to know more about this, and so I would keep clicking and read a little bit more” (P6).

**Access to information on a lot of topics (breadth of topics)**

- “[It would help if the platform had], like, a more robust portfolio of different like classes and articles” (P4).

**Consistent care or support from the same people over time on the digital platform**

- “The sleep coach that I am talking to now. She’s the same one I’ve talked to every single time...[It’s important to have continuity], especially just like to not have to answer the same questions over and over again, like they just kind of know who I am, looking for me, my style, and just more get to the care rather than have to do the basic information each session” (P3).

**Proactive outreach from the digital resource (pushes content to me; provider reaches out to me)**

- “One thing I like about the Baby Center or a lot of the articles I get from other providers is that they’re just in the email, and I don’t have to go anywhere” (P7).

**Care or content that fits with my culture and identity**

- “The [online doctor] knew Indian culture. So they knew Indian body like how our body reacts after [giving birth]. We are different than other people. So by giving specific things or a suggestion to Indians that is specific to my body, like after delivery, Indians don’t drink too much water. They understand, but my [in-person] doctor will say, drink four litre five litre today. I know so but one of the doctor on Maven when I talk, she said, I know the Indian people don’t drink too much water just after delivery, so I understand that. So if you want to have less water, so you can have a coconut water instead of normal water that will increase the hydration. So that kind of suggestion I don’t get from normal doctor” (P2).

Others said that they trusted digital content that was reviewed by medical professionals (Pilot P3) or had a “provider stamp of approval” (P9). A couple of participants were looking for digital resources that provided information from the people who conducted the research (P8) and that included “the latest research and peer reviewed evaluation” (P3).

In interviews, participants explained that access to free digital health services enables users to consume more health resources and spend more time interacting with health care providers than during in-person appointments:

[When there are] no limit on appointments...suppose I forgot something and I want another appointment and I can do it today. I don’t have to wait for another appointment.

P2

They also accessed types of resources that they valued but would not use if there was an out-of-pocket cost:

So I think I just wouldn’t have gotten what I needed [in-person] because I have two other children, and I didn’t [get what I needed] with them. Just, like you know, Google things or Pinterest, or talk to friends, and that’s it. But because this service was free to me I felt like “well, it’s there, and I might as well use it,” so I’ve taken advantage of it. And so things like a sleep consultant I probably wouldn’t have paid for. But it was just so nice to have a sounding board...So yeah, I think just having services there that I wouldn’t have made the effort to use before, but because they’re just sitting there on my phone, I might as well, and it’s free to me.

P3

Participants highlighted that free digital resources are particularly important as the cost of in-person services is very high and they were looking for a cheaper alternative:

Free resources—that’s big, since in person visits are so expensive.

P5

Interview participants also wanted to interact with digital health content and people who provided information and support without judgment. A participant explained that it is easier to receive nonjudgmental care from a digital health care provider as the person only knows the information that is being shared in the moment and cannot be biased by previous interactions or conditions. Another participant explained that she did not want to discuss her baby’s sleep issues with her in-person pediatrician as she was worried that the physician would judge her as a bad mother. It felt safer to explore the problem with the web-based provider, with whom she did not have an ongoing relationship (Pilot P4).

Interview participants shared anecdotes illustrating the accessibility of web-based providers and described connecting with these providers at night and on weekends or around their work or family schedules:

Whereas in person, they’re always busy. So there’s some waiting time...If I had concerns, I want to consult with somebody...[it’s] very helpful where I can just find someone to talk to within the week, sometimes even the same day...Being able to get in touch with someone quickly during pregnancy and even postpartum has been super helpful.

P5

There was one time where I had a concern and it was 2AM and I reached out to a practitioner over the phone, it might have been with Maven or it might have been with Telehealth, and [I had] flexibility with getting help at abnormal times of day.

P8

In general, the importance of digital health platform features was consistent by race, parity, and mental health. Differences emerged for free resources, which were more important to respondents without a mental health history compared with those with a mental health history (*P*=.02), and consistent care from the same people over time on the digital platform (*P*=.01) and access to information on a lot of topics (*P*=.02) were more important for Hispanic and people of racial and ethnic minorities respondents than for non-Hispanic White respondents.

For both pregnant and postpartum survey respondents, the 2 least important features were care or content that fit with their culture and identity and proactive outreach from the digital resources. These features were selected as extremely important by less than 20% of respondents. In interviews, participants described experiences on digital health platforms that were enhanced by resources and support that were respectful of and relevant to their culture or identity. When specifically asked about the importance of digital health resources that “take their whole identity into account,” participants consistently interpreted this to be about race, ethnicity, and gender or sexual identity, and participants from non–historically marginalized populations tended to report that inclusion was not relevant for them. This emerged in comments such as “Personally, I’m a woman, heterosexual, so I fit in fine, more than fine” (Pilot P1) and “[Health care is] not hard for me to navigate as a straight, White woman” (Pilot P3). However, as they shared anecdotes about their use of digital health platforms, they described experiences that were meaningfully inclusive (or noninclusive) related to other characteristics, such as their lifestyle (eg, family structure, work choices, and how they cared for their child) or religious practices. A participant appreciated that a nutritionist took the time to accommodate the fact that she kept kosher and “went above and beyond to research kosher supplements and make sure they were appropriate” for her to take (Pilot P2), and a working mother described her appreciation for resources that recognized her more limited availability to be with her children:

I’m probably in the majority, for most of the like, you know, bubbles you would fill in of race, gender, like I’m not a minority in any of those, but I think that everyone was just very respectful...maybe because they got to just like learn a little bit about me.

P3

People of racial and ethnic minorities participants appreciated when there were racially diverse providers on the platform. Even though the participants did not necessarily choose to interact with a member of their ethnicity or race, they valued the presence of diversity:

I felt included throughout the process. I think even seeing the diversity of the people you can choose for a specific topic also helps just, like, feel more included, like just her background. I am Mexican American. So I was born in the US but Mexican background. And so just seeing the diversity on the team was important to me.

P8

## Discussion

### Principal Findings in Relation to Prior Research

This mixed methods study found that the reasons why perinatal people use digital resources are consistent with previous research. During pregnancy, our survey and interview participants were highly interested in fetal development [10,11,16,18,20,21], reassurance about what is normal [5,16,17,38], and childbirth [10,11,20,21,39], although in our study, the latter topic was not among the top reasons for use. Unlike other studies, we found less interest in health and nutrition during pregnancy. During the postpartum period, our results were concordant with those of previous research with regard to users having a high interest in feeding, especially breastfeeding; developmental milestones; and the baby’s health [18,19,21]. Although interest in infant sleep is also common in the literature [18,19], we did not explicitly ask about this. However, this need is consistent with our overall finding that respondents were looking for information about caring for their baby and with anecdotes in our interviews that centered on infant sleep when participants discussed wanting to know what is normal, reaching out to digital providers at night, and receiving nonjudgmental support. We found an especially high interest in using digital resources for breastfeeding help among respondents with a mental health history (17/20, 85%), which is consistent with studies showing an association between maternal anxiety and breastfeeding outcomes [40-42]. Aligning with findings from content analyses of perinatal forums, top interests included baby-related topics and pain during pregnancy [17,19], but we found less interest in using digital resources for the health of the birthing person, and we did not ask about relationships (a common topic in web-based perinatal forums [17,19]) as this did not emerge as a key interest during the formative interviews. In addition to finding many of the same known reasons for using digital resources, we found that using these resources to help manage anxiety or depression was commonly selected as extremely important (55/147, 37.4% during pregnancy and 45/110, 40.9% during the postpartum period), especially for people of racial and ethnic minorities during pregnancy (21/35, 60%).

Our survey identified that, during the postpartum period, reasons for using digital resources related to the health of the birthing person were ranked lower than reasons related to obtaining information or help about the baby. This aligns with findings that mothers tend to focus on their babies and deprioritize their own needs [43]. However, there are physical and emotional challenges during the fourth trimester that often go unaddressed and can negatively affect maternal and child outcomes [44,45]. Contributing to underuse is a misalignment between the issues being addressed by providers and the birthing person’s needs, discomfort with discussing “embarrassing” issues with providers and feeling judged by them, and not knowing where to access information [43,46,47]. Digital health resources provide an opportunity for birthing people to be more in control of when, how, and for which needs they access self-care resources. Even though self-care may not be the primary priority for using digital health resources, making these supports available is valuable for those times when the birthing person is ready and interested in using them.

Emerging from both our surveys and interviews was the preference for using digital health resources to access providers. The characteristics that make digitally based providers helpful are differentiators from in-person care, with providers on platforms being reported as spending more time, focusing on issues and questions that matter to the user, and providing support quickly and conveniently [48-50]. Although study participants were explicitly asked about what is important to them when using digital health resources in general, they all had access to Maven, which offers access to a wide range of provider services. Study participants may have been more likely to express the importance of digitally based providers as they had the opportunity to use them during their perinatal stage. Users of digital health services who do not have access to digitally based providers may not identify them as important as they have not had the opportunity to experience their value. Although our results may not replicate those among digital health users who have not had access to these providers, this does not mean that digitally based providers are not an important part of what should be offered in digital health. Rather, it is an opportunity to meet a need that people may not realize they have.

Generally, the needs and preferences for perinatal digital resource use did not show much variation by perinatal phase (pregnancy vs postpartum), parity, demographics, or health characteristics. The least important reasons for use were the same during pregnancy and the postpartum period: help with healthy eating and being physically active (and, for the postpartum period, help with losing weight), connecting with a community of people in the same perinatal stage, and obtaining recommendations of things to buy to care for the birthing person or baby. Platform features were ranked in generally the same order during pregnancy and the postpartum period. However, there seems to be a difference in the overall importance of information during pregnancy versus the postpartum period, with reasons for using digital resources being selected as extremely important by a greater percentage of respondents during pregnancy than post partum. During pregnancy, the only reasons for using digital resources that differed by race were non–pregnancy-related (help with managing mental health and physical health problems), which may reflect gaps in in-person care for people of racial and ethnic minorities [51,52]. In interviews, we found a difference in participants who said that care or content that fits with their cultural, racial, or ethnic identity was important to them, with people of racial and ethnic minorities describing the value of being able to access providers with a shared identity or who provided culturally relevant support, whereas non-Hispanic White participants tended to say that this was not relevant to them. We were surprised that we did not find this difference in the surveys. A possible reason for this discrepancy is that our survey analysis focused on “extremely important” responses, and it may be that culturally relevant support is important but not extremely important. This would align with comments in the interviews about liking that providers of the same race were available even if they did not choose to use these providers.

This study included a national sample from the United States. Although our sample underrepresented people who identify as Black and Hispanic and lower-income people, it includes a significant portion of sustained users of digital resources (based on their completion of questionnaires on the Maven platform at multiple time points throughout pregnancy and the postpartum period), which is valuable as they represent people who will often use digital resources and have the potential to be significantly helped and supported by them. However, future research should also explore these topics among lower-frequency users to identify and understand barriers or deficiencies in the resources that may be contributing to reduced use.

This study adds to existing research by linking survey responses to other pregnancy, health, and demographic data about the respondents. Psychological states and mental health, especially perinatal anxiety, in relation to digital use are understudied [20], as are race and ethnicity. This formative research found only minor differences in digital resource needs and preferences across several user characteristics, including perinatal phase, parity, demographics, and mental and chronic health conditions. If this finding holds up in additional research, it has implications for the design of digital resources as it suggests that it may be appropriate to cover the same high-level perinatal topics for users across these groups. However, additional research is needed to better understand whether the content within these topics needs to be tailored to different populations. For example, future research could find that, although it is appropriate to include resources on childbirth for all users, there may be a need for more information about birthing centers in resources targeting Black pregnant people. Additional insights that are needed to improve the relevance and impact of these resources include understanding the use of the internet by nonbirthing partners [20] and the impact of use on clinical outcomes and user satisfaction [51]. There is also an opportunity to develop a standardized set of methods and metrics for assessing perinatal digital resources [4].

### Limitations

This study has some limitations. First, the generalizability of these results is limited as all survey and interview participants had access to private insurance, the sample had a relatively high income, and Hispanic and Black respondents were underrepresented. Second, there was a high amount of missingness within our race and ethnicity data from respondents selecting that they prefer not to provide this information (17/110, 15.5% and 35/147, 23.8% for postpartum and pregnancy survey respondents, respectively). This missingness may limit the reliability of our results regarding differences in digital needs by race. Finally, not all survey respondents completed the Maven questionnaires, and those who did not complete at least one Maven questionnaire were excluded from the analyses of the survey. This potentially biased the sample toward heavier users of digital resources, although this creates an opportunity to understand use needs among this segment of users.

## Appendix

Multimedia Appendix 1 Digital health platform features identified as extremely important during pregnancy by parity, mental health status, race, and ethnicity (N=147).

Multimedia Appendix 2 Digital health platform features identified as extremely important during the postpartum period by parity, mental health status, race, and ethnicity (N=110).

## Abbreviations

IRB: institutional review board

## Data Availability

Owing to the sensitive nature of the questions asked in this study, respondents were assured that raw data would remain confidential but may be shared upon request.
