# Supplementary material for: Digital Health Needs and Preferences During Pregnancy and the Postpartum Period: Mixed Methods Study
Source: JMIR Form Res. 2024 Jan 12;8:e48960. doi: 10.2196/48960 (PMC10818239; doi:10.2196/48960)
Supplement: Multimedia Appendix 1 [file formative_v8i1e48960_app1.pdf]

Digital health platform features identified as *extremely important* during pregnancy by parity, mental health status, and race and ethnicity (N=147)

		Parity			Mental Health			Race and ethnicity*		
**Digital health platform features**	**Overall (N=147 n(%))**	**Parous (n=50) n(%)**	**Nulliparous (n=97) n(%)**	**p-value**	**No mental health history (n=105) n(%)**	**Mental health history (n=37) n(%)**	**p-value**	**Non-White (n=35) n(%)**	**Non-Hispanic, White (n=77) n(%)**	**p-value**
Credible and trustworthy information and providers	117 (79.6)	35 (70.0)	82 (84.5)	0.18	83 (79.0)	29 (78.4)	1.00	23 (65.7)	64 (83.1)	0.55
Non-judgmental information/support	97 (66.0)	29 (58,9)	68 (70.1)	0.40	68 (64.8)	24 (64.9)	1.00	21 (60.0)	53 (68.8)	1.00
Digital resources that are free to me	89 (60.5)	24 (48.0)	65 (67.0)	0.09	70 (66.7)	16 (43.2)	0.02	19 (54.3)	49 (63.6)	1.00
Fast access to appointments	75 (51.0)	22 (44.0)	53 (54.6)	0.48	56 (53.3)	17 (45.9)	0.64	17 (48.6)	39 (50.6)	0.75
Easy to find information; easy to navigate	74 (50.3)	26 (57.8)	48 (51.6)	0.62	54 (54.5)	17 (50.0)	0.80	18 (62.1)	35 (47.3)	0.26
Access to appointments at convenient times	72 (49.0)	24 (48.0)	48 (49.5)	0.99	53 (50.5)	17 (45.9)	0.88	16 (45.7)	39 (50.6)	0.99
Information that is actionable (specific recommendations for what to do)	64 (43.5)	23 (46.0)	41 (42.3)	0.55	48 (45.7)	14 (37.8)	0.59	17 (48.6)	34 (44.2)	0.35
Receive fast responses to my digital messages	59 (40.1)	20 (40.0)	39 (40.2)	0.92	44 (41.9)	13 (35.1)	0.67	15 (42.9)	26 (33.8)	0.19
Resources that are specific to my needs (personalized)	49 (33.3)	15 (30.0)	34 (35.1)	0.86	38 (36.2)	9 (24.3)	0.30	14 (40.0)	23 (29.9)	0.16
Access to a lot of information on each topic (depth of information)	36 (24.5)	11 (22.0)	25 (25.8)	0.92	29 (27.6)	6 (16.2)	0.27	10 (28.6)	14 (18.2)	0.16
Access to information on a lot of topics (breadth of topics)	35 (23.8)	11 (22.0)	24 (24.7)	1.00	30 (28.6)	5 (13.5)	0.11	12 (34.3)	13 (16.9)	0.02
Consistent care or support from the same people over time on the digital platform	31 (21.1)	9 (18.0)	22 (22.7)	0.79	26 (24.8)	5 (13.5)	0.24	11 (31.4)	9 (11.7)	0.01

Proactive outreach from digital resource (pushes content to me, provider reaches out to me)	26 (17.7)	6 (12.0)	20 (20.6)	0.36	21 (20.0)	4 (10.8)	0.31	8 (22.9)	8 (10.4)	0.07
Care or content that fits with my culture and identity	26 (17.7)	10 (20.0)	16 (16.5)	0.64	19 (18.1)	6 (16.2)	1.00	6 (17.1)	10 (13.0)	0.55

* Respondents who selected “I prefer not to say” for race and ethnicity were not included in this comparison.
