# Supplementary material for: Digital Health Needs and Preferences During Pregnancy and the Postpartum Period: Mixed Methods Study
Source: JMIR Form Res. 2024 Jan 12;8:e48960. doi: 10.2196/48960 (PMC10818239; doi:10.2196/48960)
Supplement: Multimedia Appendix 2 [file formative_v8i1e48960_app2.pdf]

Digital health platform features identified as *extremely important* during postpartum by parity, mental health status, and race/ethnicity (N=110)

		Parity			Mental Health			Race and ethnicity*		
**Digital health platform features**	**Overall (N=110) n(%)**	**Parous (n=14) n(%)**	**Nulliparous (n=58) n(%)**	**p-value**	**No mental health history (n=49) n(%)**	**Mental health history (n=20) n(%)**	**p-value**	**Non-White (n=21) n(%)**	**Non-Hispanic, White (n=57) n(%)**	**p-value**
Credible and trustworthy information and providers	82 (74.5)	12 (85.7)	46 (79.3)	0.60	39 (79.6)	16 (80.0)	1.00	15 (71.4)	44 (77.2)	0.90
Digital resources that are free to me	60 (54.5)	12 (85.7)	29 (50.0)	0.11	29 (59.2)	11 (55.0)	1.00	10 (47.6)	34 (59.6)	0.91
Non-judgmental information/ support	58 (52.7)	9 (64.3)	30 (51.7)	1.00	25 (51.0)	12 (60.0)	0.49	11 (52.4)	31 (54.4)	0.86
Information that is actionable (specific recommendations for what to do)	57 (51.8)	7 (50.0)	33 (56.9)	0.44	25 (51.0)	14 (70.0)	0.12	11 (52.4)	30 (52.6)	0.76
Fast access to appointments	52 (47.3)	8 (57.1)	29 (50.0)	1.00	23 (46.9)	11 (55.0)	0.56	9 (42.9)	29 (50.9)	1.00
Easy to find information; easy to navigate	53 (48.2)	9 (64.3)	24 (41.4)	0.44	22 (44.9)	9 (45.0)	1.00	10 (47.6)	26 (45.6)	0.67
Access to appointments at convenient times	50 (45.5)	7 (50.0)	24 (41.4)	1.00	19 (38.8)	9 (45.0)	0.69	8 (38.1)	26 (45.6)	1.00
Receive fast responses to my digital messages	40 (36.4)	7 (50.0)	20 (34.5)	0.72	18 (36.7)	9 (45.0)	0.57	8 (38.1)	17 (29.8)	0.41
Resources that are specific to my needs (personalized)	33 (30.0)	5 (35.7)	17 (29.3)	1.00	13 (26.5)	8 (40.0)	0.32	4 (19.0)	20 (35.1)	0.38
Consistent care or support from the same people over time on the digital platform	33 (30.0)	5 (35.7)	13 (22.4)	0.51	11 (22.4)	6 (30.0)	0.63	5 (23.8)	18 (31.6)	1.00
Access to a lot of information on each topic (depth of information)	22 (20.0)	3 (21.4)	12 (20.7)	1.00	10 (20.4)	4 (20.0)	1.00	5 (23.8)	7 (12.3)	0.15

Access to information on a lot of topics (breadth of topics)	22 (20.0)	2 (14.3)	13 (22.4)	0.49	9 (18.4)	6 (30.0)	0.38	5 (23.8)	8 (14.0)	0.28
Care or content that fits with my culture and identity	14 (12.7)	3 (21.4)	6 (10.3)	0.40	5 (10.2)	4 (20.0)	0.25	3 (14.3)	6 (10.5)	0.68
Proactive outreach from digital resource (pushes content to me, provider reaches out to me)	12 (10.9)	2 (14.3)	3 (5.2)	0.30	4 (8.2)	1 (5.0)	1.00	3 (14.3)	4 (7.0)	0.35

* Respondents who selected “I prefer not to say” for race and ethnicity were not included in this comparison.
